# Two Types of Medical Leader

**Published:** 1917-10-06

**Authors:** 


### TWO TYPES OF MEDICAL LEADER.

### No. I.—The Hero.

By CADUCEUS.

OF what fine quality his mind was one never saw better than at the first meeting; never, perhaps, so well. Here was a professor giving lectures in temporary quarters in the West End of London to thirty or forty young men, most of them ex-civil surgeons back from the South African War, their pockets distended with accumulated pay, their minds with a consciousness of having survived peril and hardship, of being launched on a life career, and of living within a few minutes of the chief metropolitan pleasure resorts. The course of tuition they were now undergoing was of minor practical importance, selfishly regarded: diligence, or want of diligence, could not affect their prospects much. Moreover, owing to special circumstances, it was abbreviated. Also everybody knew that this was the last batch of military medical aspirants that would listen to the lecturer, who had resigned his appointment for another at less than half the salary. They found, too, that the most of what he taught was nothing likely to be useful, at least nothing of general acceptance, but just his own ideas; while, when a certain disease was the subject, and a certain worker whom the text-books honoured for discovery in connection with it was prominent in the front seats, was most clearly visible, even to spectacled eyes, from the dais, the discourse made no more mention of him than of the porter at the door. No, the hero was not a slave to tact, even in the case of those he respected. "So I said to Ehrlich, 'Who is the drunken fool who has said this?'" And Ehrlich replied, 'I am the drunken fool.'" A strange category to calculate, is cause and effect! For on top of all this the class, who slept through or "cut" many of the allocutions delivered by the hero's colleagues, who assisted at the burning of very little midnight oil except at supper-rooms, who had the stupendous weight on their minds of getting a first uniform, really talked about nothing so much as—who could have imagined it?—these self-same irrelevant theories. They believed them, lock, stock, and barrel, with the immoderation common in youth, only an immoderation rare of its direction. They would go up to him at the end of a lecture—"Sir, why don't other people know all this?" and get the conversationally spoken answer that one gave these things to an unbelieving world. How they growled when they heard a few words of mild criticism on him from one of the colleagues! How they smiled when they heard that analysis of the water in his cistern had shown a large amount of a chemical very prominent in his investigations! They actually regretted the opportunities of previous classes, who had had a full quota of lectures, the last one dealing with philosophy (being half-educated Britons, they said "religion" instead). They talked of the traditions of the farewell dinner, where he was chaired round the room, being first considerately given time to remove his spectacles. Something in this kind was attempted one evening. "Have a drink, sir!" "Yes, sir, do—a liqueur gentian violet!" "Well, you see, that's the worst of me, I don't drink. But I'll have a bun." So he was bought, and ate, a bun.

The class passed on, more quickly than most classes do, but with much more regret tempering the joy of advance, and even put some of the theories into practice. An obscure private soldier, knowing little how many rich people would in a few years' time pay heavily for the same privilege, had his back quickly cleared of chronic boils; whereupon his colonel, with the love of uniformity that marks the combatant mind (remember the admiral who would have the ship's band slope all their fiddlesticks at the same angle), wanted the rest of the regiment injected. I fancy a good deal of calcium lactate was prescribed for people sick of very diverse disorders. Older scholars were met with, and their traditions concerning the hero picked up. These narratives, these *ana*, though of varying accuracy, were still good evidence of the impression he made. It was said that he had gained a scholarship in a non-medical subject, and devoted it to the study of medicine; that at least once he had given himself a disease in order to investigate it; that all his ideas were "awfully clever, but not much use"; that he would experiment on his children; that his walking-stick had to be tied to his wrist, or he would lose it. . . . On the whole he seemed unpopular only with the lazy, pleasure-loving ones; or with those who thought, if they didn't speak, of ward duty as "coming down to doing clinical work," who conversed in the clipped tones, the smiling gabble of the cavalryman.

Then there was an interval of two or three years, and he seemed to fill the medical journals as he had once filled the mouths of his class. It was as good as a play to see eminent persons getting pat with the old familiar-sounding jargon. Indeed, it was rather like being at the first night of a fine play at a London theatre after assisting at its successful trial production in America or the provinces. And the next thing was that a real play on the subject was made. There was a dramatist who came into general prominence about the same time. Now, two medical men happened to meet, one of whom knew the hero and the other the dramatist. They decided that for many reasons and purposes, one of which (unhappily unfulfilled) was the removal of certain errors from the dramatist's mind, it would be a great thing were their two distinguished friends to make each other's acquaintance. This being contrived successfully, the result was a play which testified brilliantly to the deep-lined favourable impression which the hero had the power of making so easily. This special power of influence caused some heart-burning. A certain teacher, fond of passing beyond his medical rubric, but decidedly old-fashioned when he did so, warned students with more or less of good-humour against the "Celtic Siren." This was unhappily said, because for one thing the hero, like so many men of talent, was of mixed race. It was not very effective either. How could a graceful scholar, who sat down as a young man to write a text-book as his chief work, and inscribed that text-book to a priest —how could a routineer like this compete for influence on the adolescent mind with a born pioneer, one in the true line of succession of Chaucer's Leech and Balzac's Despleins? Although young men are in general bad critics, they were right enough here.

Well, that is the main history of the hero. He still does work that perhaps no one else could do, but it is not like those first ideas. With the onset of middle age, as the scholar aforesaid has well argued, imagination is bound to die down. Now he has to rely upon his intellect alone; his style of writing gets crabbed, too. To be candid, in all his work I see a trace of justification for that robust criticism of a nonentity—"not much use." At first sight it (not he) promises so much that performance has to lag behind. The good his example has done to students is incalculable. An utterly uncommercial knowledge-getter, or, rather, which is much rarer, knowledge-diviner, he must have destroyed again and again the ideals of popularity and profit, and replaced them with the spirit of original scientific inquiry. For many a medical mind he has set up a right standard by which to size and sort the many claimants to attention. This alone is much to have done, for if love of the best is "the fiend that man harries," love of the second-rate is the fiend that, intellectually speaking, nullifies him and his.

